# Supplementary material for: Regulatory T cells inhibit CD8+ TRM-like cells during the early stages of tumor immune escape
Source: bioRxiv. 2025 Oct 22:2025.10.21.683143. Preprint. [Version 1] doi: 10.1101/2025.10.21.683143 (PMC12633329; doi:10.1101/2025.10.21.683143)

bioRxiv preprint doi: https://doi.org/10.1101/2025.10.21.683143; this version posted October 22, 2025. The copyright holder for this preprint (which was not certified by peer review) is the author/funder, who has granted bioRxiv a license to display the preprint in perpetuity. It is made available under aCC-BY 4.0 International license.

Supplemental figure legends

Supplementary Figure S1 | Multiplex imaging panel validation and immune cell composition in human stage II melanoma. **A**, Overview of the 21-marker t-CyCIF antibody panel used to identify immune and stromal cell populations, annotated by lineage. **B**, Heatmap showing scaled (z-score) expression of lineage and functional markers across single-cell clusters identified from multiplex imaging data. $CD8^+$ T cells are stratified by CD103 expression, and $CD4^+$ T cells by Foxp3 expression. **C** and **D**, Relative proportions (**C**) and absolute cell numbers (**D**) of immune, stromal, and melanoma cell populations across all samples. **E**, UMAP plots colored by samples and $CD8^+CD103^-$, $CD8^+CD103^+$, $CD4^+Foxp3^-$, and $CD4^+Foxp3^+$ T cell populations. **F**, Distribution plot showing Treg to $CD8^+CD103^+$ T cell distances. Significance was determined by a Mann-Whitney U rank test was in **F**.

Supplementary Figure S2 | T_{RM}-like $CD8^+$ TIL frequencies in engraftable versus autochthonous melanoma models and OT-I cell proliferation. **A**, Representative flow cytometry plots of CD8 TILs expressing CD103 and CD69 in tumors from BPO-derived cell lines engrafted s.c. and their corresponding primary autochthonous BPO tumors. **B**, Average frequencies of T_{RM}-like $CD8^+$ TILs in primary autochthonous tumors versus s.c. engrafted tumors. BPO primary (autochthonous) tumors were taken at the time of s.c. engraftable experiments and are not the primary BPO-derived cell line tumors. **C**, OT-I proliferation in TdLN after transfer into tumor bearing or tumor-free BPO mice. Graphs display all mice from two (**A** and **B**) or one (**C**) independent experiment. Significance was determined by a Kruskal-Wallis test with Dunn's multiple comparison correction in **B**.

Supplementary Figure S3 | Histopathology of early-stage tumors. **A**, Representative H&E images showing hyperpigmented lesions confined to upper dermis.

Supplementary Figure S4 | Stability of T_{RM}-like $CD8^+$ TIL niche during tumor progression. **A**, Representative flow cytometry plots showing CD103 and CD101 expression in early- and late-stage tumors. **B**, Representative flow cytometry plots and cell counts of T cells in peripheral blood after 30 days of FTY720 treatment. Graphs display all mice from 2 independent experiments. Significance was determined by a Mann-Whitney U rank in **A** and a Kruskal-Wallis test with Dunn's multiple comparison correction in **B.**

Supplementary Figure S5 | Immune-focused t-CyCIF antibody panel and melanoma biopsy quantification. **A**, Antibody panel composition for immune cell subset identification in mouse t-CyCIF. **B**, Expression of markers for cell phenotyping. **C** and **D,** Relative proportion of immune cell and stromal cell subsets across time points (**C**) or individual mice (**D**).

Supplementary Figure S6 | $Sox10^+$ melanoma cell localization in pre-pigmentation stage. **A**, Representative t-CyCIF image showing $Sox10^+$ melanoblast localization to lower hair follicle bulb. **B**, Scattered dermal and papillary dermis $Sox10^+$ melanoma cells (white arrows) in pre-pigmentation biopsies.

Supplementary Figure S7 | Heterogeneity of immune microenvironments in early-stage hyperpigmented melanoma cell clusters. **A**-**C**, Representative H&E and t-CyCIF images highlighting immune cell composition in hyperpigmented lesions. Sites i-ii, v, and vi show the presence of immune cell clustered with $Sox10^+$ melanoma cells. Clusters with high TRM-like cell density and abundant granzyme B expression. Sites iv-vi exemplify Treg cell enrichment in some hyperpigmented areas. Sites iv and vii, exemplify macrophage-dominant clusters with sparse $CD8^+$ T cells. Inset in site vii shows a single $Gzmb^+$ T_{RM}-like cell in contact with a Treg cell.

Supplementary Figure S8 | $CD8^+$ T_{RM}-like cell tumor specificity and role in immunoediting.

bioRxiv preprint doi: https://doi.org/10.1101/2025.10.21.683143; this version posted October 22, 2025. The copyright holder for this preprint (which was not certified by peer review) is the author/funder, who has granted bioRxiv a license to display the preprint in perpetuity. It is made available under aCC-BY 4.0 International license.

A, H-2K^b/SIINFEKL pentamer staining showing tumor antigen specificity in T_{RM}-like versus CD103$^-$ CD8$^+$ TILs at early and late stages of tumor development. **B**, Experimental schematic for CD8$^+$ T cell depletion starting at pigmentation onset.**C**, Confirmation of CD8$^+$ TIL depletion at endpoint. **D**, Frequency of EGFPOVA$^+$ tumor cells in nontreated and CD8-depleted versus immunodeficient (BPO/Rag1$^-$/$^-$) mice. Significance was determined by a Kruskal-Wallis test with Dunn's multiple comparison correction was used in **A** and **D** and a Mann-Whitney U rank test in **C**.

Supplementary Figure S9 | Changes in immune cell composition after Treg depletion. A, Frequencies of CD4$^+$ T cells, γδ T cells, dendritic cells, macrophages, NK cells, and neutrophils after systemic Treg depletion. **B**, Recruitment of Foxp3$^-$ CD4$^+$ Tconv cells after systemic Treg depletion ± FTY720. **C**, Tim-3 expression on CD8$^+$ TILs after systemic Treg depletion ± FTY720. **D**, Tim-3 expression on CD8$^+$ TIL subsets after local (DT i.t.) Treg depletion. **E**, OT-I T cell counts in TdLN after Treg depletion with prior anti-CD8β antibody treatment. Graphs display all mice from 2-3 independent experiments. Significance was determined by a Kruskal-Wallis test with Dunn's multiple comparison correction in **A-D**.

Supplementary Figure S10 | Flow cytometry gating strategy. Separate gates were used to isolate lymphocytes and tumor cells. Tumor cells were identified to be within the CD45$^-$ SSChi fraction. Cell populations were identified as follows; Neutrophils: CD45$^+$ SSChi MHCII$^-$ CD24int Ly6G$^+$, Macrophage: CD45$^+$ MHCII$^+$ CD11b$^+$ CD24$^-$ CD11c$^-$, cDC1: CD45$^+$ MHCII$^+$ CD24$^+$ CD11b$^-$ CD11c$^+$ XCR1$^+$, gd T cell: CD45+ SSClow TCRb$^-$ TCRgd$^+$, NK cell: CD45$^+$ SSClow TCRb$^-$ TCRgd$^-$ NK1.1$^+$, CD8 T cell: CD45$^+$ SSClow TCRb$^+$ CD8a$^+$, CD4 T cell: CD45$^+$ SSClow TCRb$^+$ CD4$^+$. Treg cells were identified as CD4$^+$ T cell with Foxp3EGFPDTR$^+$. TRM-like cells were identified as CD8$^+$ T cell with CD103 expression.

bioRxiv preprint doi: https://doi.org/10.1101/2025.10.21.683143; this version posted October 22, 2025. The copyright holder for this preprint (which was not certified by peer review) is the author/funder, who has granted bioRxiv a license to display the preprint in perpetuity. It is made available under aCC-BY 4.0 International license.

Figure S1

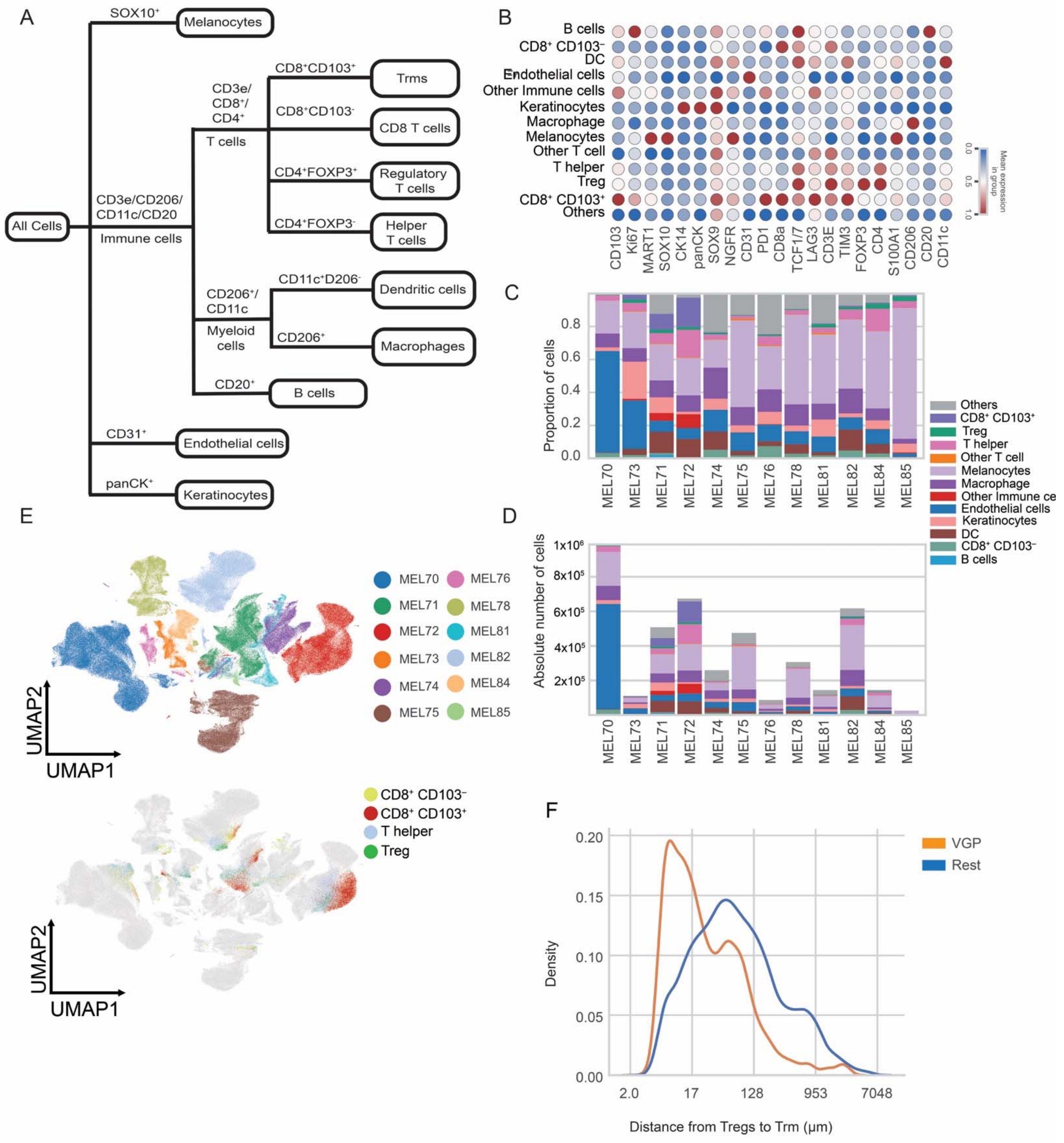

bioRxiv preprint doi: https://doi.org/10.1101/2025.10.21.683143; this version posted October 22, 2025. The copyright holder for this preprint (which was not certified by peer review) is the author/funder, who has granted bioRxiv a license to display the preprint in perpetuity. It is made available under aCC-BY 4.0 International license.

Figure S2

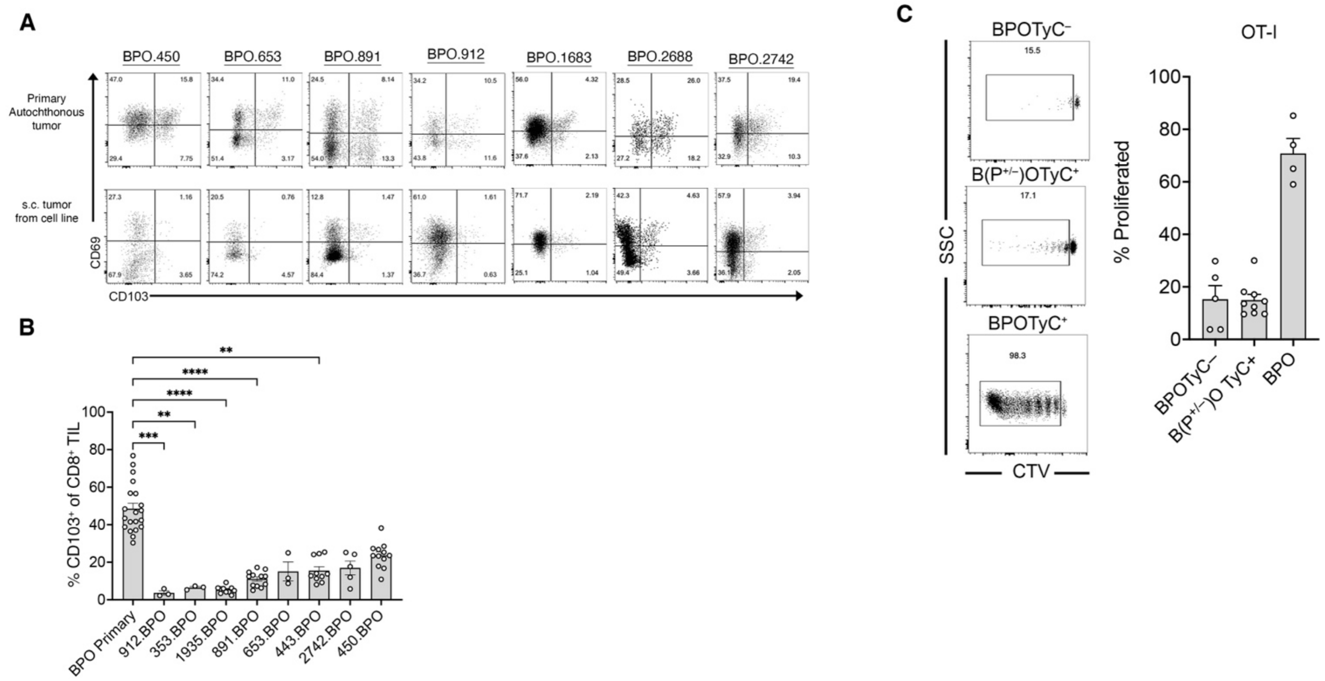

bioRxiv preprint doi: https://doi.org/10.1101/2025.10.21.683143; this version posted October 22, 2025. The copyright holder for this preprint (which was not certified by peer review) is the author/funder, who has granted bioRxiv a license to display the preprint in perpetuity. It is made available under aCC-BY 4.0 International license.

Figure S3

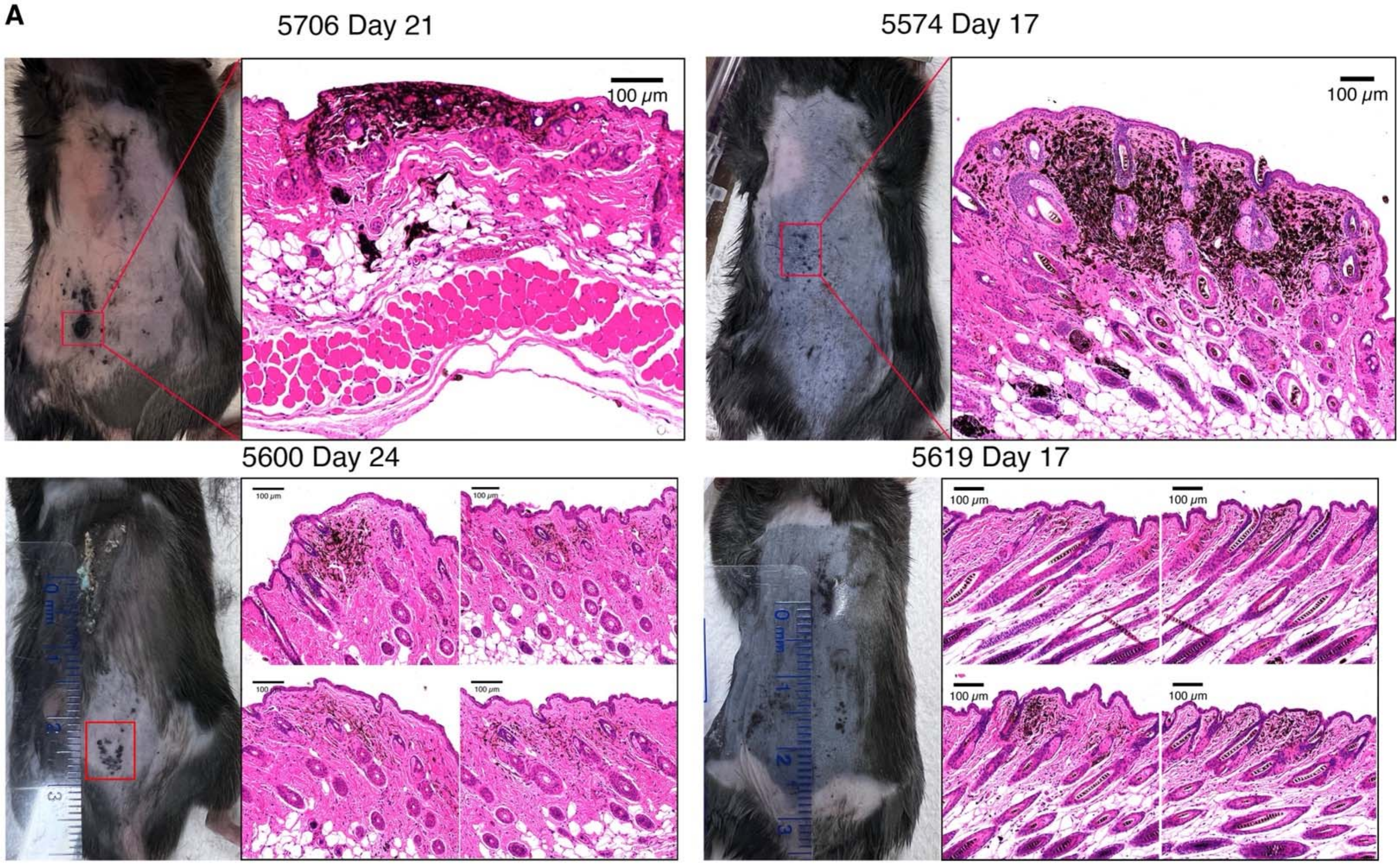

bioRxiv preprint doi: https://doi.org/10.1101/2025.10.21.683143; this version posted October 22, 2025. The copyright holder for this preprint (which was not certified by peer review) is the author/funder, who has granted bioRxiv a license to display the preprint in perpetuity. It is made available under aCC-BY 4.0 International license.

Figure S4

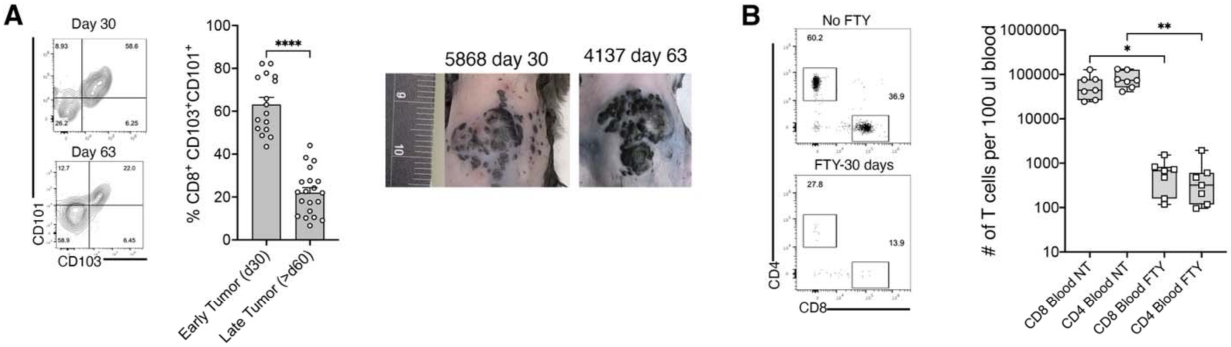

bioRxiv preprint doi: https://doi.org/10.1101/2025.10.21.683143; this version posted October 22, 2025. The copyright holder for this preprint (which was not certified by peer review) is the author/funder, who has granted bioRxiv a license to display the preprint in perpetuity. It is made available under aCC-BY 4.0 International license.

Figure S5

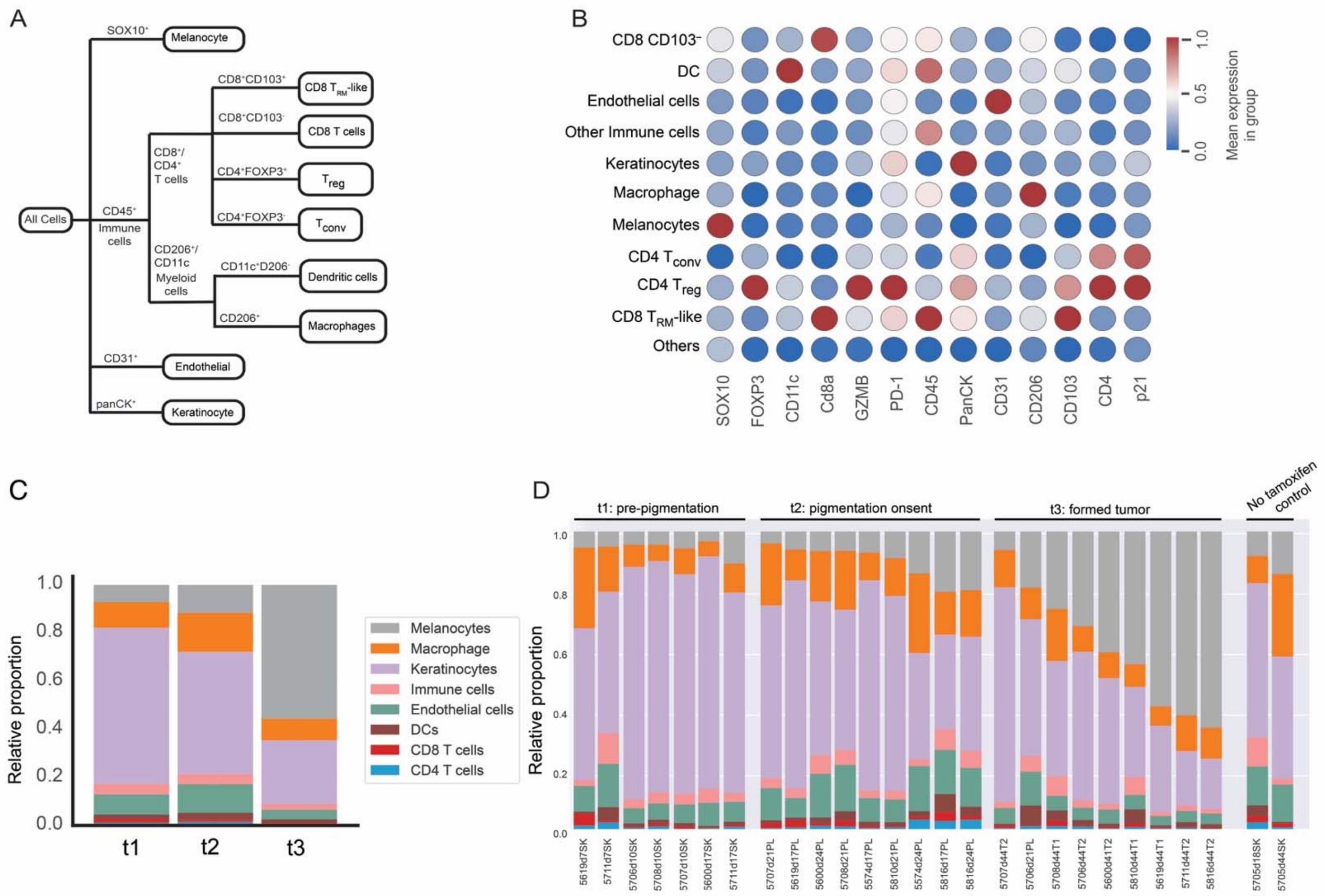

bioRxiv preprint doi: https://doi.org/10.1101/2025.10.21.683143; this version posted October 22, 2025. The copyright holder for this preprint (which was not certified by peer review) is the author/funder, who has granted bioRxiv a license to display the preprint in perpetuity. It is made available under aCC-BY 4.0 International license.

Figure S6

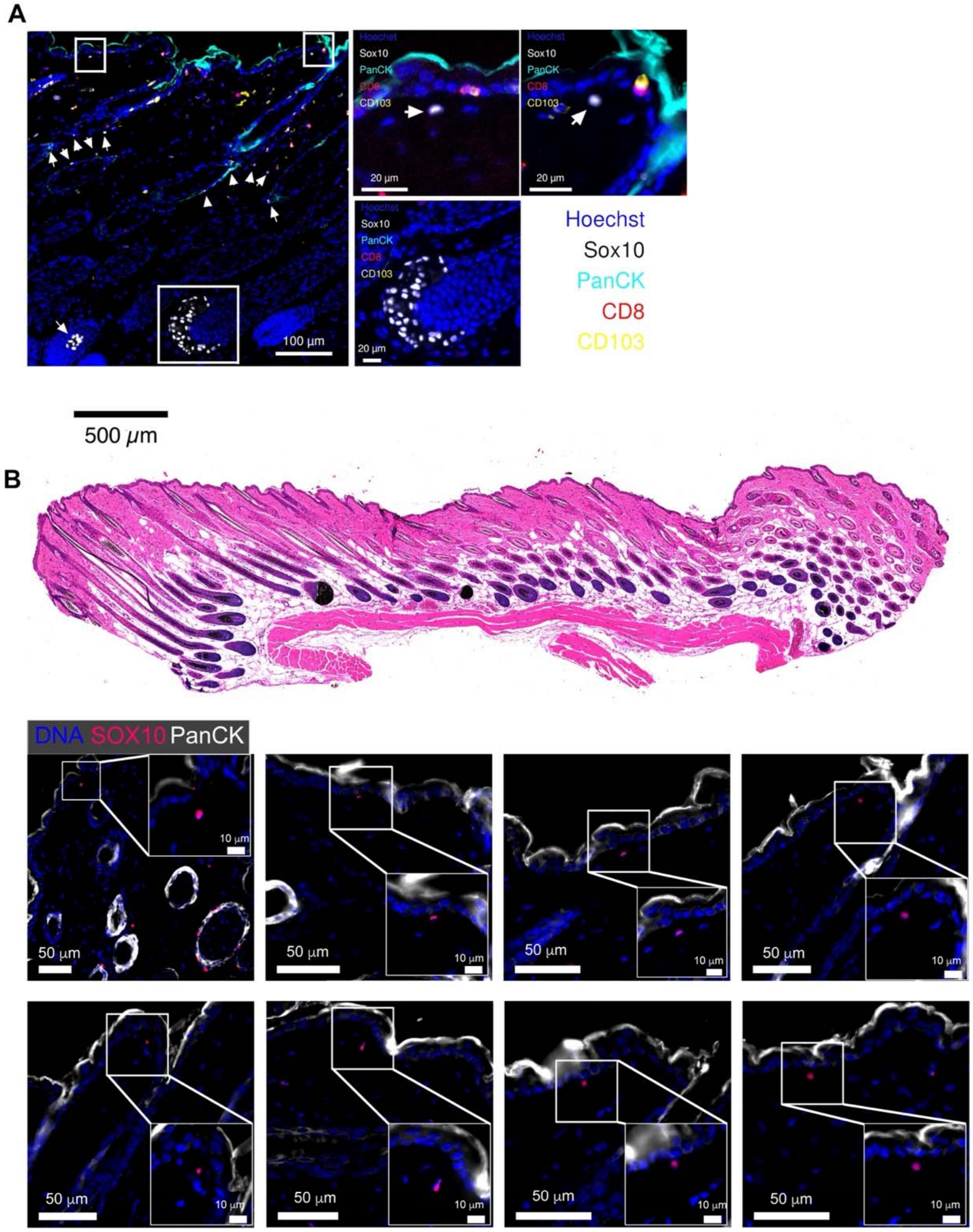

bioRxiv preprint doi: https://doi.org/10.1101/2025.10.21.683143; this version posted October 22, 2025. The copyright holder for this preprint (which was not certified by peer review) is the author/funder, who has granted bioRxiv a license to display the preprint in perpetuity. It is made available under aCC-BY 4.0 International license.

Figure S7

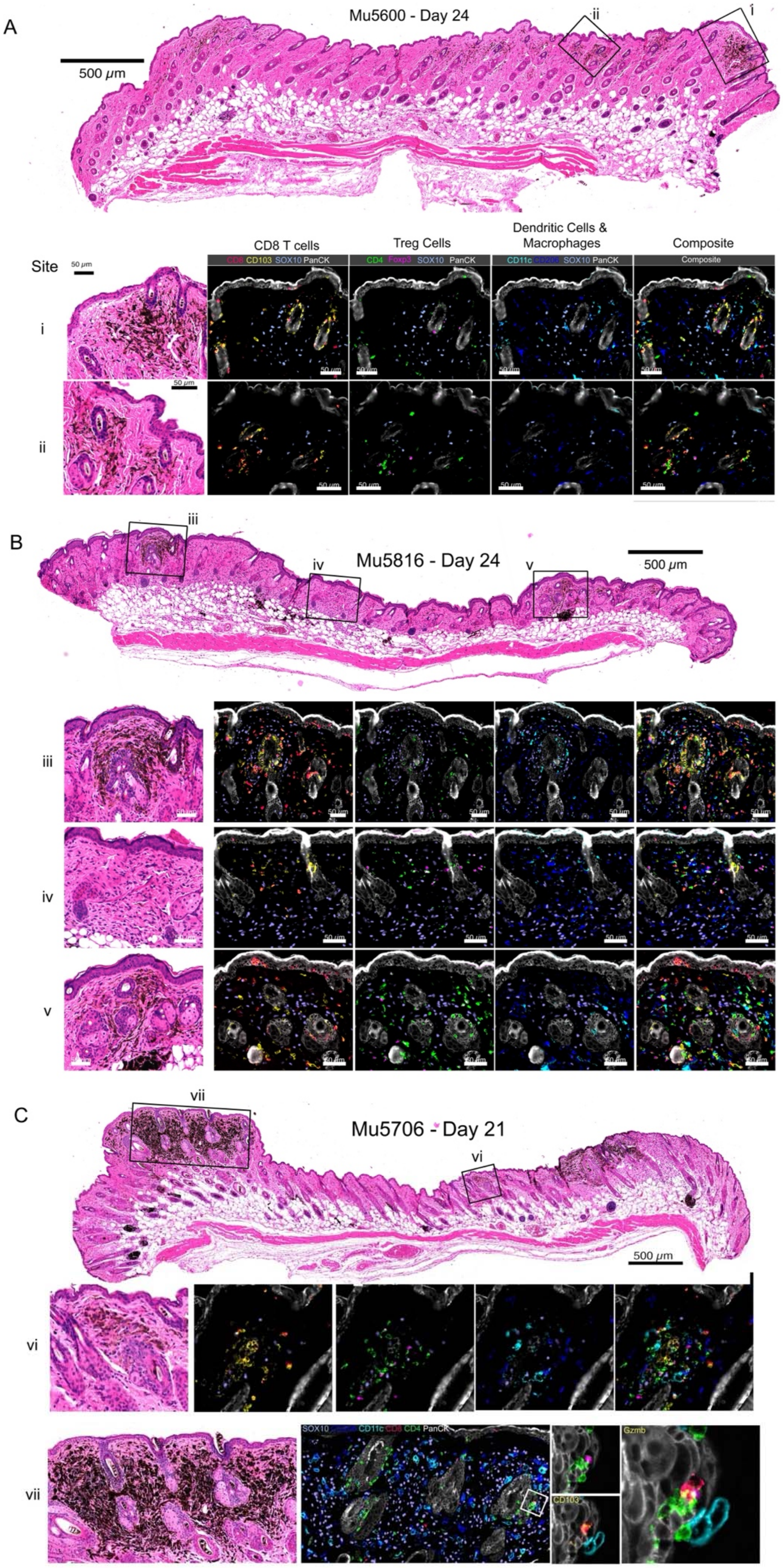

bioRxiv preprint doi: https://doi.org/10.1101/2025.10.21.683143; this version posted October 22, 2025. The copyright holder for this preprint (which was not certified by peer review) is the author/funder, who has granted bioRxiv a license to display the preprint in perpetuity. It is made available under aCC-BY 4.0 International license.

Figure S8

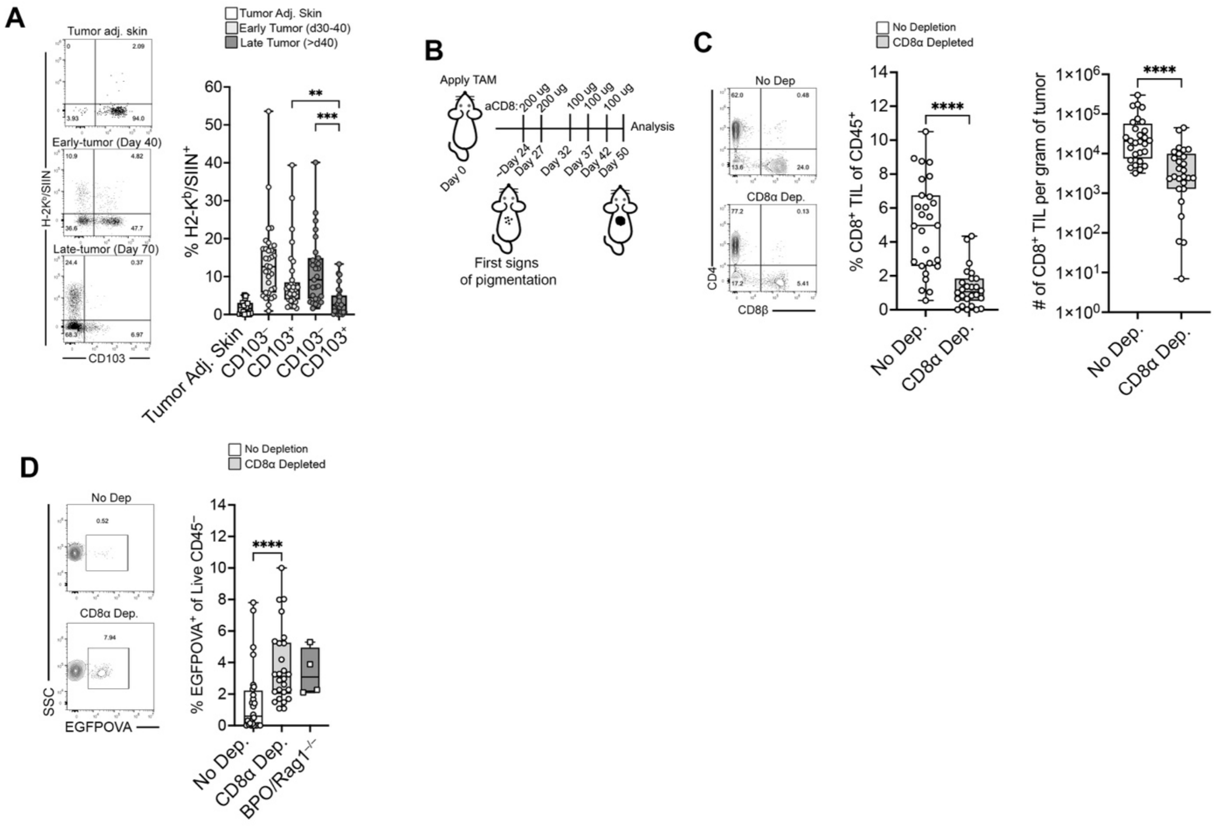

bioRxiv preprint doi: https://doi.org/10.1101/2025.10.21.683143; this version posted October 22, 2025. The copyright holder for this preprint (which was not certified by peer review) is the author/funder, who has granted bioRxiv a license to display the preprint in perpetuity. It is made available under aCC-BY 4.0 International license.

Figure S9

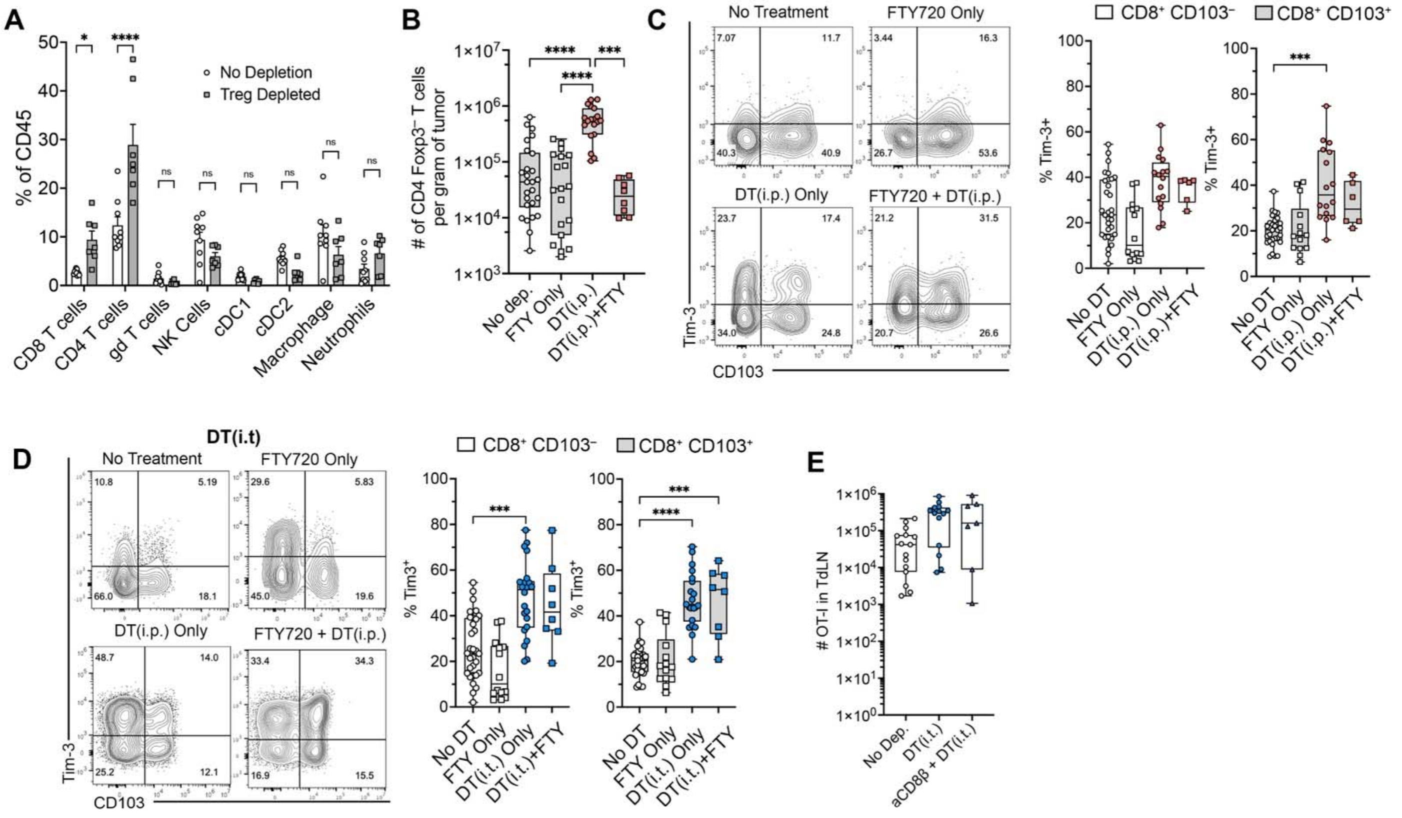

bioRxiv preprint doi: https://doi.org/10.1101/2025.10.21.683143; this version posted October 22, 2025. The copyright holder for this preprint (which was not certified by peer review) is the author/funder, who has granted bioRxiv a license to display the preprint in perpetuity. It is made available under aCC-BY 4.0 International license.

Supplemental Figure 10

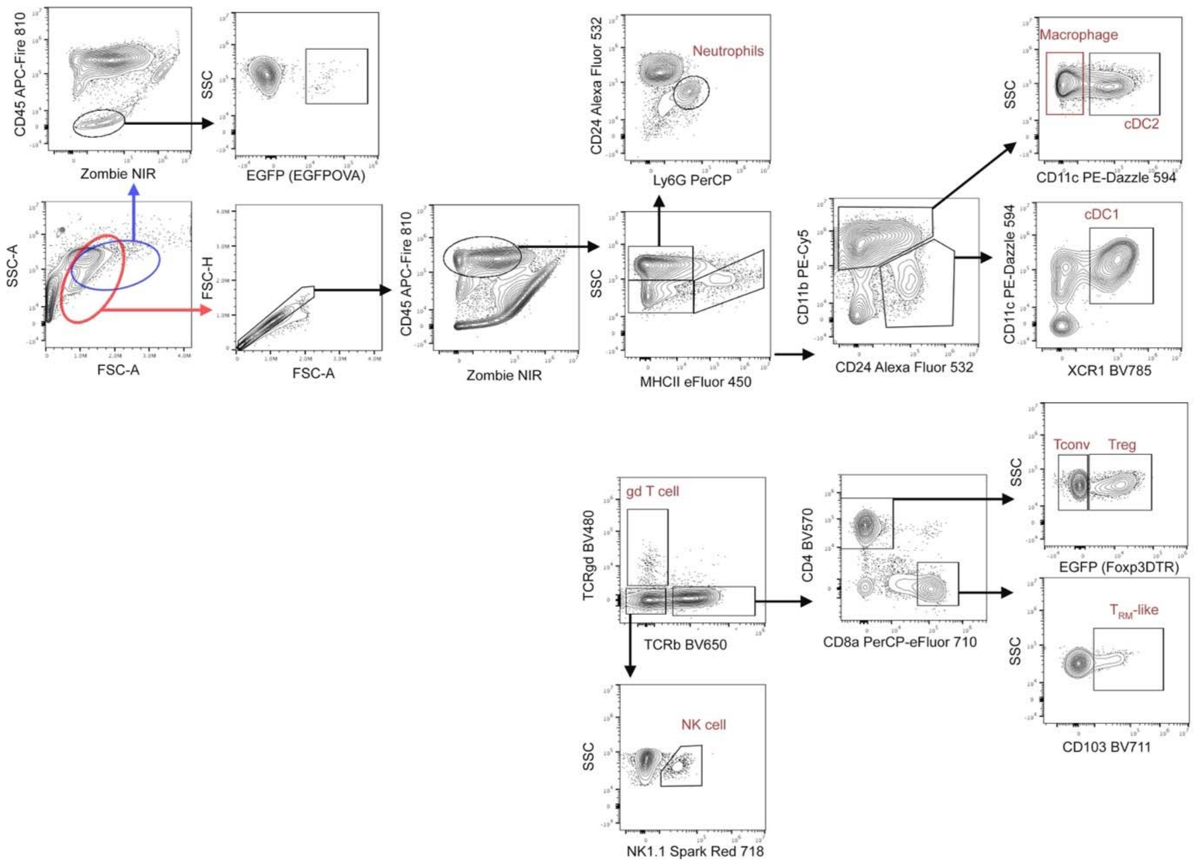

Supplement: 1 [file NIHPP2025.10.21.683143V1-supplement-1.pdf]
